# Recovery, restoration, and risk: a cross-sectional survey of the impact of COVID-19 on GPs in the first UK city to lock down

**DOI:** 10.3399/BJGPO.2020.0151

**Published:** 2020-12-02

**Authors:** Namrata Trivedi, Vivek Trivedi, Arumugam Moorthy, Hina Trivedi

**Affiliations:** 1 Final Year Medical Student, Imperial College London, London, UK; 2 CT1 Doctor, Royal Albert Edward Infirmary, Wrightington, Wigan and Leigh NHS Trust, Wigan, UK; 3 International Visiting Professor, TNDr MGR Medical University, Chennai, India; 4 Consultant Rheumatologist, University Hospitals of Leicester NHS Trust, Leicester, UK; 5 Honorary Associate Professor, College of Life Sciences, Leicester Medical School, University of Leicester, Leicester, UK; 6 Honorary Diabetes Fellow, Leicester Diabetes Centre, University of Leicester, Leicester, UK; 7 GP Senior Partner, Horizon Healthcare, Leicester, UK; 8 Honorary Teaching Fellow, Leicester Medical School, University of Leicester, Leicester, UK

**Keywords:** COVID-19, coronavirus, mental health, personal protective equipment, general practice

## Abstract

**Background:**

The COVID-19 pandemic has impacted GPs immensely. Work patterns have changed, risk stratification has been proposed, and the mental health of clinicians has been adversely affected. The COVID-19 prevalence among GPs is unknown. This study focuses on assessing the impact of COVID-19 on GPs in Leicestershire, the first UK city to lock down locally.

**Aim:**

This survey assessed the prevalence of COVID-19 in GPs and explored GP work patterns in comparison with national guidance. It used a validated perceived stress tool to evaluate the impact of COVID-19 on GP stress perception.

**Design & setting:**

The cross-sectional retrospective survey was sent to all the GPs in Leicestershire.

**Method:**

A total of 111 GPs in Leicestershire took part voluntarily in an anonymised questionnaire-based study. A 29-item survey using SmartSurvey software was designed with multiple choice and Likert response scale questions.

**Results:**

COVID-19 prevalence in GPs in Leicestershire was 8.1%; 70.3% of GPs were of Black, Asian, and minority ethnic (BAME) origin; 91.9% of GPs had undergone risk stratification; and 79.3% of GPs felt supported by their practice, but only 59.5% felt supported with mental health. GPs described feeling more stressed during the COVID-19 pandemic than they had been previously.

**Conclusion:**

This is the first study evaluating COVID-19 prevalence among GPs in Leicestershire. Despite government interventions, GPs felt less supported with their mental health compared with pre-COVID-19 times. Thus, the NHS in England should focus on GP stress and wellbeing as they work towards the restoration and recovery of primary care while battling the second wave.

## How this fits in

The prevalence of COVID-19 among UK GPs is unknown and this study attempted to assess the prevalence in Leicestershire. This study highlighted a change in GP work patterns and implementation of risk stratification. GPs already on the threshold of burnout are at greater risk. The worsening mental wellbeing in the GP cohort during COVID-19 suggests the need for further support during the second wave of the pandemic.

## Introduction

COVID-19 has been classified as an international pandemic^1^ causing significant morbidity, mortality, and huge financial burden on society.^2^ The UK passed its peak infection rate in the first wave,^3^ and commenced a recovery and restoration phase prior to the current second wave. GPs have radically changed their work patterns during this period.^4^

On 19 March 2020, a letter from NHS England asked all GP practices to adopt a full triage model supporting remote patient management.^5^ Additionally, NHS England asked GP practices to manage home visits in designated premises and set up video consultations.^5^

Concurrently, as the COVID-19 death toll climbed, some hospitals reported 44% of their staff testing positive for COVID-19.^6^ The prevalence of COVID-19 among GPs was not well documented. Characteristics of patients who were high-risk were identified,^7^ showing a disproportionate death rate among patients from BAME groups.^8^ Importantly, the Office for National Statistics reported that the BAME population is up to four times more likely to die from COVID-19.^9^ Consequently, Public Health England (PHE) issued guidance on risk assessing healthcare professionals (but alarmingly did not include recommendations to mitigate risks for the high-risk BAME NHS workers).^10^ Nevertheless, local workplaces adopted pragmatic modifications to work patterns.^11^

Additionally, this pandemic and the added isolation of a national lockdown has caused undue mental stress,^12^ with increased suicide rates in the general population.^13^ Perceived stress is the culmination of individual feelings or thoughts about how much stress one is under at a given point or time period.^14^ Thus, GPs who are already known to have a stressful vocation^15^ may be prone to worsening mental health issues as a result of this pandemic.

The aim of this cross-sectional survey was to assess the prevalence of COVID-19 among GPs in Leicestershire, the first area of local lockdown, and evaluate its impact on their working patterns and stress levels, not previously investigated at the time of writing. GP mental wellbeing was explored using the Perceived Stress Scale.^16^

## Method

This was a cross-sectional, retrospective questionnaire-based study. GPs across Leicestershire were invited to take part voluntarily.

A 29-item survey using SmartSurvey software was designed with multiple choice and Likert response scale questions. The validated Perceived Stress Scale^16^ was used to evaluate stress prior to COVID-19 (March 2020) and currently at the time of survey circulation (July–August 2020). Responses regarding stress levels prior to COVID-19 and during the COVID-19 pandemic were completed during the time of the study period.

The survey was distributed through the local GP global email to eliminate selection bias, and responses were anonymous. The study period was from 24 July 2020–7 August 2020. The completed responses were then analysed using the online SmartSurvey software and Microsoft Excel.

## Results

A total of 185 participants accessed the survey. Seventy-one incomplete responses and three responses from non-GPs were excluded. Responses from 111 GPs were subsequently included for analysis (see Supplementary Appendix S1 and S2). Twenty-seven responders added comments (see Supplementary Appendix S2).

Sociodemographic characteristics of the participants (Table 1) revealed 51.4% of responders were male and 96.4% were aged <65 years. With regard to ethnic group, 23.4%, 70.3%, and 5.4% of GPs were British, BAME, and other, respectively, with 0.9% not disclosing ethnicity. It was found that 56.8%, 15.3 %, 20.7%, and 5.4% were GP partners, locums, salaried GPs, and GP trainees, respectively. In addition, 65.8% of GPs had >10 years experience and 73.0% of practices were teaching practices.

**Table 1.** Sociodemographic profile of GP cohort, *N* = 111

**Characteristics**	**Responses, *n* (%)**
**Sex**	
Male	57 (51.4)
Female	52 (46.8)
Prefer not to say	2 (1.8)
**Age, years**	
25–34	16 (14.4)
35–54	66 (59.5)
55–64	25 (22.5)
65–74	3 (2.7)
≥75	1 (0.9)
**Ethnic group**	
British	26 (23.4)
Black, Asian, minority ethnic	78 (70.3)
Other	6 (5.4)
Did not want to disclose	1 (0.9)
**Type of GP**	
GP trainee	6 (5.4)
Salaried GP	23 (20.7)
Locum GP	17 (15.3)
Partner	63 (56.8)
Other	2 (1.8)
**Experience as G** **P** **, years**	
Still in training	6 (5.4)
<2	6 (5.4)
2–5	15 (13.5)
6–10	11 (9.9)
>10	73 (65.8)
**Registered teaching practice**	
Yes	81 (73.0)
No	28 (25.2)
I don’t know	2 (1.8)

### Prevalence of COVID-19 in Leicestershire GPs

**Figure 1.**
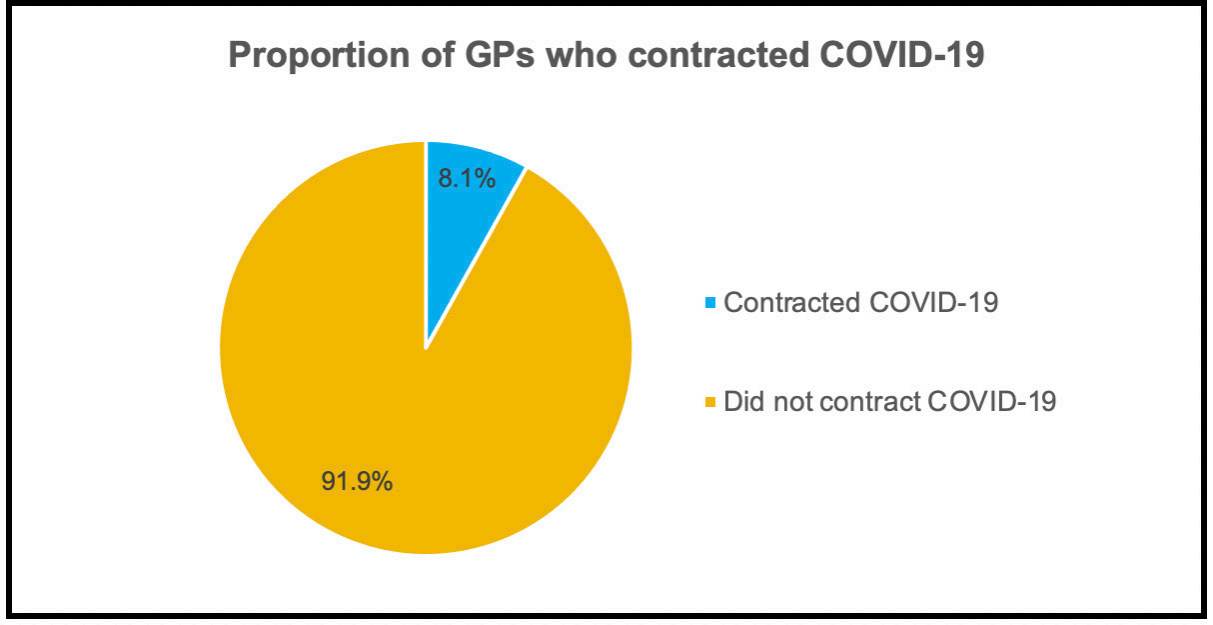
Pie chart showing the proportion of GPs who contracted COVID-19 compared with those who did not, *N* = 111

It was found that 8.1% (*n* = 9) of responders had self-reported that they had contracted COVID-19 compared with 91.9% (*n* = 102) who did not (Figure 1).

### Changes in work pattern

For home visits, 86.5% (*n* = 96) of responders stated that they had decreased, and 98.2% (*n* = 109) reported a reduction in face-to-face consultations. While 91.0% (*n* = 101) and 97.3% (*n* = 108) responded that video and telephone consultations had increased, respectively, with a 48.6% (*n* = 54) reported increase in email consultations (Table 2).

**Table 2.** Changes in work pattern, *N* = 111

**Changes in work pattern**	**Increased, ***n* (%)****	**Stayed the same, *n* (%)**	**Decreased, *n* (%)**
**Home visits**	2 (1.8)	13 (11.7)	96 (86.5)
**Face-to-face consultations**	0 (0.0)	2 (1.8)	109 (98.2)
**Video consultations**	101 (91.0)	10 (9.0)	0 (0.0)
**Telephone consultations**	108 (97.3)	1 (0.9)	2 (1.8)
**Email consultations**	54 (48.6)	56 (50.5)	1 (0.9)

### Personal protective equipment (PPE)

It was found that 16.2% (*n* = 18) of GPs had ‘poor’ availability of and accessibility to PPE, with 40.5% (*n* = 45) reporting this to be ‘average’ or ‘neutral’, while 43.3% (*n* = 48) rated this as ‘good’ or ‘excellent’ (data not shown).

### Risk stratification

For risk stratification, 91.9% (*n* = 102) of participants had undergone this process with 71.2% (*n* = 79) of these suggesting the implementation of this risk stratification was ‘good’ or ‘excellent’ (data not shown).

### Perceived Stress Scale

Participants reported an increased frequency of perceived stress across all 10 domains of the Perceived Stress Scale during the COVID-19 pandemic (‘currently’), compared with pre-COVID-19 times (Table 3).

**Table 3.** Responses to the Perceived Stress Scale questions, *N* = 111

**Perceived Stress Scale questions**	**Before COVID-19 and currently**	**Responses, % (*n***)
***Never***	***Almost never***	***Sometimes***	***Fairly often***	***Very often***	***Always***	***Don’t know***
**Feeling upset because of something that happened unexpectedly**	**Pre-COVID-19**	7.2 (8)	28.8 (32)	43.2 (48)	10.8 (12)	9.9 (11)	0.0 (0)	0.0 (0)
	**Currently**	9.0 (10)	13.5 (15)	36.0 (40)	19.8 (22)	17.1 (19)	4.5 (5)	0.0 (0)
**Feeling confident about your ability to handle your personal problems**	**Pre-COVID-19**	1.8 (2)	3.6 (4)	9.0 (10)	17.1 (19)	34.2 (38)	30.6 (34)	3.6 (4)
	**Currently**	2.7 (3)	3.6 (4)	16.2 (18)	24.3 (27)	24.3 (27)	27.0 (30)	1.8 (2)
**Feeling unable to control the important things in life**	**Pre-COVID-19**	14.4 (16)	25.2 (28)	36.9 (41)	13.5 (15)	9.0 (10)	0.0 (0)	0.9 (1)
	**Currently**	12.6 (14)	13.5 (15)	24.3 (27)	19.8 (22)	24.3 (27)	2.7 (3)	2.7 (3)
**Finding that you cannot cope with all the things you had to do**	**Pre-COVID-19**	18.0 (20)	28.8 (32)	27.9 (31)	10.8 (12)	10.8 (12)	3.6 (4)	0.0 (0)
	**Currently**	13.5 (15)	24.3 (27)	25.2 (28)	16.2 (18)	18.9 (21)	1.8 (2)	0.0 (0)
**Feeling nervous and 'stressed'**	**Pre-COVID-19**	12.6 (14)	17.1 (19)	41.4 (46)	20.7 (23)	8.1 (9)	0.0 (0)	0.0 (0)
	**Currently**	9.0 (10)	9.0 (10)	24.3 (27)	27.0 (30)	21.6 (24)	8.1 (9)	0.9 (1)
**Being able to control irritations in your life**	**Pre-COVID-19**	1.8 (2)	5.4 (6)	10.8 (12)	25.2 (28)	29.7 (33)	25.2 (28)	1.8 (2)
	**Currently**	0.9 (1)	9.0 (10)	24.3 (27)	23.4 (26)	24.3 (27)	16.2 (18)	1.8 (2)
**Feeling that things were going your way**	**Pre-COVID-19**	1.8 (2)	6.3 (7)	10.8 (12)	33.3 (37)	33.3 (37)	12.6 (14)	1.8 (2)
	**Currently**	3.6 (4)	12.6 (14)	24.3 (27)	28.8 (32)	18.0 (20)	10.8 (12)	1.8 (2)
**Feeling that you were on top of things**	**Pre-COVID-19**	0.0 (0)	7.2 (8)	9.0 (10)	27.0 (30)	39.6 (44)	16.2 (18)	0.9 (1)
	**Currently**	0.0 (0)	11.7 (13)	18.0 (20)	23.4 (26)	34.2 (38)	11.7 (13)	0.9 (1)
**Being angered because of things that were outside of your control**	**Pre-COVID-19**	11.7 (13)	30.6 (34)	40.5 (45)	11.7 (13)	2.7 (3)	2.7 (3)	0.0 (0)
	**Currently**	9.0 (10)	23.4 (26)	22.5 (25)	22.5 (25)	17.1 (19)	5.4 (6)	0.0 (0)
**Feeling that difficulties were piling up so high that you could not overcome them**	**Pre-COVID-19**	20.7 (23)	37.8 (42)	27.0 (30)	8.1 (9)	5.4 (6)	0.9 (1)	0.0 (0)
	**Currently**	17.1 (19)	27.0 (30)	25.2 (28)	16.2 (18)	12.6 (14)	1.8 (2)	0.0 (0)

It was found that 20.7% (*n* = 23) more GPs reported they more often felt 'upset with something that happened unexpectedly' during the COVID-19 pandemic compared with pre-COVID-19 times. Similarly, 24.3% (*n* = 27) and 27.9% (*n* = 31) more responders stated they more often felt they were 'unable to control the important things in life' and felt 'nervous and stressed', respectively, currently compared with pre-COVID-19.

Also, 21.6% (*n* = 24) fewer GPs felt 'things were going their way' compared with pre-COVID-19 times, while 27.9% (*n* = 31) more GPs reported they were more often 'angered because of things outside of their control' during this pandemic compared with pre-COVID-19 times.

### Support from the workplace

**Figure 2.**
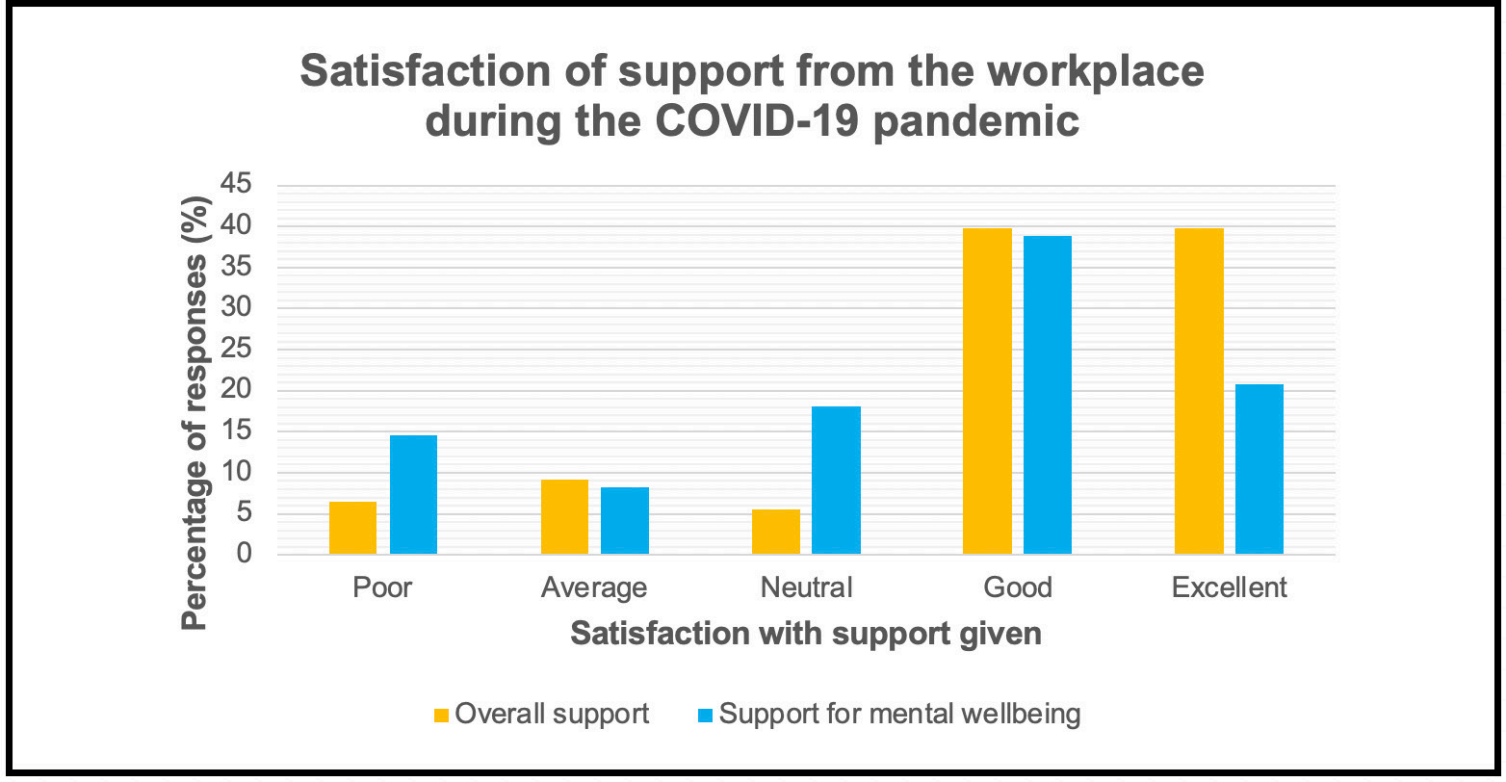
Bar chart showing the difference in satisfaction of overall support given from the workplace and support towards the mental wellbeing of GPs*,* *N* = 111

Figure 2 shows that 79.3% (*n* = 88) of GPs reported their overall support from their workplace was ‘good’ or ‘excellent’. While 6.3% (*n* = 7) of GPs stated it was ‘poor’, whereas 14.4% (*n* = 16) stated this was ‘average’ or ‘neutral’ during the COVID-19 pandemic.

For support with mental wellbeing, 14.4% (*n* = 16) of participants stated it was ‘poor’, 26.1% (*n* = 29) ‘average’ or ‘neutral’, and 59.5% (*n* = 66) ‘good’ or ‘excellent’ during the COVID-19 pandemic.

In readiness for the second wave, 62.2% (*n* = 69) of responders stated the preparation was ‘good’ or ‘excellent’.

### Qualitative comments

Responders included the following remarks: *'* *The CCG* [clinical commissioning group] *and clinical lead input and leadership has been non* *-* *existent and a disgrace in Leicester* *'*; and *'* *General anxiety levels higher* *—* *mainly because of dealing with uncertainty* *or* *worry at lack of back-up from secondary care, worry about coping over winter with inevitable increasing pressures* *.* *'* Other comments included: *'* *considerable stress in these difficult times* *'*; *'* *local lockdown had a bigger negative effect on my perception* *'*; and *'* *the NHS should be ashamed of how they handled the situation* *'*.

## Discussion

### Summary

The study found 8.1% of the responding GPs had contracted COVID-19, with most responders being BAME. GP work involved more technology-based consultations (Table 2) and 91.9% of all GPs had undergone risk stratification. Consequently, 71.2% of GPs rated implemented changes to reduce their risk as ‘good’ or ‘excellent’. Importantly, 79.3% of GPs felt supported by their practice and 62.2% stated ‘good’ or ‘excellent’ preparation for the second wave. However, only 43.3% rated the access and availability to PPE as ‘good’ or ‘excellent’, suggesting room for improvement in providing this for the second wave. Strikingly, only 59.5% felt supported with mental health, with the validated stress tool indicating that GPs were stressed more often during the COVID-19 pandemic compared with the pre-COVID-19 time. Several qualitative comments were included in the results. This indicates that further support is required for GPs for this second wave of the pandemic.

### Strengths and limitations

A major strength of this first cross-sectional survey involving a large proportion of participants from BAME backgrounds was that it assessed the prevalence of COVID-19 among GPs within the first local lockdown area. Importantly, this study assessed the mental wellbeing of GPs, who have already been described to be at risk of burnout.^15^ GPs are known to be poor responders to surveys;^17^ therefore, this sample size may be reasonable. However, a future survey incorporating a larger radius would greatly strengthen the study. Limitations include the qualitative nature of this survey. A high proportion of BAME GPs responded, which may include bias in the results for mental wellbeing because of the current evidence on the adverse impact of COVID-19 on BAME doctors.^8^ Additionally, specific diagnostic criteria for COVID-19 were not included in the survey. Self-reporting may result in inaccurate prevalence estimations. Another limitation was that this study was conducted in a city that was in an extended lockdown which — although it provides evidence for the impact of lockdown — may not be representative of the total GP population. Lastly, the design of this survey and potential ambiguity of some questions may have caused confusion in responses. This study will aid the development of better-designed surveys in the future.

### Comparison with existing literature

The prevalence of COVID-19 among the GPs in this survey was higher than the national average (326 000 cases in 66.65 million, which equates to 0.05% of the UK population).^18,19^ This increased prevalence is likely a result of GPs being on the frontline in primary care. This may also reflect the increased testing from the local Leicestershire lockdown. Other contributory factors may be the large BAME proportion of responders in Leicestershire,^20^ as ethnic group has been linked to COVID-19,^8^ and that 40.1% of the medical force in the NHS are BAME.^21^

The results from this survey suggest that video and telephone consultations are the new way of working. The technology-based consultations have superseded the previous norm of face-to-face consultations and home visits (Table 2). These findings were in line with the NHS England guidance released in March 2020.^5^ Since then, the British Medical Association (BMA)^22^ and NHS England^23^ have produced clear guidance reiterating that remote consultations should be used when appropriate and home visits should be limited. Interestingly, the King’s Fund had produced a model on using digital technology and video consultations in 2018.^24^ Ironically, GPs who were resistant to accept this at the time,^25^ have now embraced this technology as a result of this pandemic.

Notably, 91.9% of the GPs in this survey had undergone risk stratification at their workplace, as mandated by NHS England,^26^ and 71.1% rated their subsequent workplace support as ‘good’ or ‘excellent’. Some providers have produced risk assessment and guidance for patients who are high-risk such as BAME staff, pregnant women, and vulnerable staff.^11^

Only 59.5% of the responders felt supported by their workplace for their mental wellbeing while 14.4% of responders felt support was ‘poor’. At baseline, one in four people suffer from mental health issues^27^ and this is likely to increase as a result of COVID-19.^12^ Despite the help from charities and the nationwide campaign with helplines to support patients and doctors alike,^28,29^ mental health issues continue to be problematic.

In addition, GPs were found to have an increase in frequency of perceived stress owing to COVID-19 across all parameters in question. These findings imply that GPs are more often exhibiting an external locus of control with the continually changing landscape of COVID-19. Evidence suggests this could lead to anger-eliciting situations and depression.^30^ Although there have been helplines for GPs to access, many will not. Previous studies have suggested GPs, in particular, are reluctant to seek help, particularly for psychological problems.^31,32^ Thus, strategies are needed to challenge this culture of self‐reliance among GPs.

### Implications for research and practice

This study provided an approximation of the COVID-19 prevalence in GPs, particularly in an area of local lockdown. Prevalence will guide the services needed to support GPs in terms of risks; for example, the degree of PPE needed and whether face-to-face consultations should be completely replaced by telemedicine.

Unfortunately, even though increased risks for BAME individuals have been highlighted by PHE and the BMA,^33^ no specific guidance has been given to BAME GPs.^10^ With these odds, should BAME GPs stop all face-to-face consultations? Without an objective risk stratification tool, consistent guidance would be difficult. The authors await national guidance on this issue, particularly for BAME GPs. Hopefully, the new UK-based study investigating COVID-19 risks for BAME healthcare staff will yield more definitive answers.^34^

The COVID-19 pandemic forced a change in working that GPs previously resisted but then swiftly adopted, as evidenced by this study. Nevertheless, future studies on satisfaction and effectiveness of these novel ways of working will be crucial to assess the success and acceptability of these virtual consultations.

Importantly, the detrimental impact of COVID-19 on GPs, who are amid the recruitment and retention crisis,^35^ implies that GPs would benefit from more support. Governmental helplines rely on GPs seeking help reactively. Despite this help, GPs still feel stressed. Given appraisal and revalidation was on hold, mentorship support was not easily available. Thus, more structured proactive support should be considered for GP mental wellbeing in order to challenge this culture of self‐reliance among GPs.

Comments regarding stress, online consultations, PPE, and the extended lockdown could also be further explored.

Future studies should include a larger cohort of GPs, with numerical variables to allow for statistical analysis, including GPs in non-lockdown areas to increase the reliability of the data retrieved. A χ^2^ test comparing the variables in pre-COVID-19 times and during COVID-19 times could be performed to obtain more valid results.

In conclusion, this study showed a higher prevalence of COVID-19 in GPs compared with the national average. The results reaffirmed the change in work patterns, mandated by NHS England. With the continual evolution of the COVID-19 pandemic, digital consultations will likely continue. However, technology-based consultations should be evaluated for effectiveness and patient satisfaction. The second wave, another national lockdown, and the increased COVID-19 prevalence is likely to magnify GP perceived stress. Thus, before the second wave peaks or any further pandemics, it is essential to support GPs in a more proactive manner as they work towards the restoration and recovery of primary care throughout this second wave.
